# Acute pressure on the sciatic nerve results in rapid inhibition of the wide dynamic range neuronal response

**DOI:** 10.1186/1471-2202-13-147

**Published:** 2012-12-04

**Authors:** Wenxue Wang, Wei Tan, Danping Luo, Jianhua Lin, Yaoqing Yu, Qun Wang, Wangyeng Zhao, Buling Wu, Jun Chen, Jiman He

**Affiliations:** 1 Department of Physiology, School of Life Science, Kuning, 605000, China; 2 Pain Medicine Program, IDD, Nanfang Hospital, Southern Medical University, Guangzhou, 510515, China; 3 Institute for Biomedical Sciences of Pain and Institute for Functional Brain Disorders, Tangdu Hospital, Fourth Military Medical University, Xi’an, 710032, China; 4 Rhode Island Hospital, Brown University, Providence, 02903, USA

**Keywords:** Sciatic nerve, WDR, Pain, Acute pressure

## Abstract

**Background:**

Acute pressure on the sciatic nerve has recently been reported to provide rapid short-term relief of pain in patients with various pathologies. Wide dynamic range (WDR) neurons transmit nociceptive information from the dorsal horn to higher brain centers. In the present study, we examined the effect of a 2-min application of sciatic nerve pressure on WDR neuronal activity in anesthetized male Sprague–Dawley rats.

**Results:**

Experiments were carried out on 41 male Sprague–Dawley albino rats weighing 160–280 grams. Dorsal horn WDR neurons were identified on the basis of characteristic responses to mechanical stimuli applied to the cutaneous receptive field. Acute pressure was applied for 2 min to the sciatic nerve using a small vascular clip. The responses of WDR neurons to three mechanical stimuli applied to the cutaneous receptive field were recorded before, and 2, 5 and 20 min after cessation of the 2-min pressure application on the sciatic nerve. Two-min pressure applied to the sciatic nerve caused rapid attenuation of the WDR response to pinching, pressure and brushing stimuli applied to the cutaneous receptive field. Maximal attenuation of the WDR response to pinching and pressure was noted 5 min after release of the 2-min pressure on the sciatic nerve. The mean firing rate decreased from 31.7±1.7 Hz to 13±1.4 Hz upon pinching (*p* < 0.001), from 31.2±2.3 Hz to 10.9±1.4 Hz (*p* < 0.001) when pressure was applied, and from 18.9±1.2 Hz to 7.6±1.1 Hz (*p* < 0.001) upon brushing. Thereafter, the mean firing rates gradually recovered.

**Conclusions:**

Our results indicate that acute pressure applied to the sciatic nerve exerts a rapid inhibitory effect on the WDR response to both noxious and innocuous stimuli. Our results may partially explain the rapid analgesic effect of acute sciatic nerve pressure noted in clinical studies, and also suggest a new model for the study of pain.

## Background

Pressure stimulation of the sciatic nerve is associated with hyperalgesia [1-3]. Recently, we found that acute pressure applied to the sciatic nerve inhibited pain [4,5]. We then proposed that pressure causing pathologies are chronic, and not acute. Long-term pressure, even at very low levels, may cause severe neural dysfunction. For example, chronic pressure applied to the sciatic nerve because of internal tension of the obturator muscle, or anatomical abnormalities in the piriformis muscle, can cause pain [6-8], and surgery to relieve pressure affords rapid relief [9-11]. On the contrary, we found that acute pressure applied to the sciatic nerve for 2 min provides rapid pain relief [4,5,12,13]. This relief is short-lived, with duration of minutes to hours. We also found that acute pressure on the sciatic nerve reduces clinical pain, but not experimental cold pressor pain [5]. The underlying mechanism is unknown.

Spinal dorsal horn WDR neurons are the first synaptic relay point for afferent pathways and they play an important role in modifying the transmission of noxious input [14]. Injuries to sciatic nerve, such as constriction, transection, etc., can cause hyperalgesic pathologies. On the other hand, sciatic nerve block can inhibit pain. Both procedures can trigger changes in WDR activity [15-17]. For example, Sotgiu at al conducted a study in rats of the background activity of WDR neurons after sciatic nerve constriction. The WDR neurons showed high frequency discharges after ligation. The increased post-injury discharges were reduced by applying lidocaine on the peripheral site of constriction [15]. Our clinical studies showed that acute pressure block on the sciatic nerve produced rapid inhibition of pain, contrary to chronic pressure, which caused sciatica. In the present study, we examined WDR activity in rats after the application of direct acute pressure on the sciatic nerve.

## Methods

### Animal preparations

Experiments were carried out on 41 male Sprague–Dawley albino rats weighing 160–280 g. The animals had access to water and food *ad libitum*, and were maintained at a temperature of 22–26°C with a light/dark cycle of 12 h. Experimental protocols were approved by the Fourth Military Medical University, People’s Republic of China. The rats were anesthetized by intra-peritoneal injection with a dose of 5 ml/kg urethane–chloralose solution (containing urethane at 250 mg/ml and chloralose at 10 mg/ml). A tracheal cannula and a left jugular vein catheter were inserted. Adequate anesthesia was confirmed intermittently during the experiment by examining the animal for spontaneous movement or whether they had an arousal response to a noxious pinch applied to the skin. The sciatic nerve was exposed high in the thigh, and was carefully isolated from the surrounding tissue. Laminectomy was performed from the T13 to L1 vertebrae to expose the lumbar enlargement for spinal neuron recording.

A pool was formed using the skin flaps surrounding the exposed sciatic nerve, and was filled with warm paraffin oil (37°C) to prevent drying. Core body temperature was monitored using a thermostat probe inserted into the rectum, and was maintained at 37.5±0.5°C using a feedback-controlled heating pad under the ventral surface of the rat’s abdomen.

### Application of pressure to the sciatic nerve and recording of WDR neuronal activity

Dorsal horn WDR neurons were identified on the basis of characteristic responses to mechanical stimuli applied to the receptive field [18-20]. Extracellular single-unit recordings were made between L4 and L6 using glass capillary micro-electrodes (10–15MV, filled with 0.5 M sodium acetate). The recording electrode was advanced in 2-μm steps using an electronically-controlled manipulator. Light stroking and probing of the skin of the ipsilateral hind paw were used as search stimuli to identify dorsal horn neurons. WDR neurons are characterized by (1) a receptive field that consists of a small low-threshold center and a large high-threshold surround; (2) an increase in firing rate in response to brushing, application of pressure and noxious pinching of the low-threshold center; (3) a response to noxious pinching (and not to the other two stimuli) by the high-threshold surround; and (4) no evident adaptation when continuous stimulation is applied to the low-threshold center.

After identification of a single WDR unit, a small vascular clip (pinch force of 100 g) was applied to the sciatic nerve for 2 min. The clip surfaces were lined with soft rubber, which absorb a considerable proportion of the pressure; the resulting pressure on the sciatic nerve was evaluated at approximately 30–70g (We measured the pressure force of the clip on the sciatic nerves during simulation by using an elastic band. The pressure force was determined by comparing deformation of an elastic strip of rubber sandwiched in the clip with that of an elastic strip of rubber sandwiched between two flat surfaces on which a series of weights were placed). Thereafter, three types of mechanical stimuli (brushing, pressure application and pinching) were delivered to the center of the receptive field. In all rats, the responses to the three mechanical stimuli were recorded before, and 2, 5 and 20 min after release of compression. Spontaneous discharges were recorded for 10 s before application of any mechanical stimulus, and these were also recorded when the vascular clip was attached to the sciatic nerve and when the clip was removed until application of the first stimulus. The mean spontaneous firing rate and the response to each mechanical stimulus were analyzed. The mean firing rate during the 20 s period before stimulation was subtracted from each response rate. The proportionate inhibition of the response to each cutaneous stimulus was also calculated; the mean firing rate upon application of each stimulus prior to compression was taken to be 100%.

The mechanical stimuli used were (1) brushing, performed by brushing of the center of the cutaneous receptive field (cRF) once per second with a hairy paint brush; (2) pressure application, performed by clamping a fold of skin between the arms of a flattened alligator clip to produce constant pressure that was not painful; and (3) noxious pinching, performed by pinching a fold of skin (with constant force) using a small serrated clip (this was painful when tested on the experimenter’s skin). The force of the flattened clip, and the serrated clip was evaluated at approximately 100–130 g and 250–280 g, respectively. For each nerve, we sequentially stimulated with brushing, pressure, and pinching for 10 s at each time point.

### Histological analysis

A vascular clip was attached to the sciatic nerve for 2 or 20 min. Thereafter, the sciatic nerve was dissected out and biopsies were taken. The specimens were fixed in buffered 4% paraformaldehyde, washed in water, dehydrated in a graded ethanol series, cleared in xylene, embedded in paraffin, and cut into 5-μm-thick cross or longitudinal sections at the compressed sites. The longitudinal and cross sections were stained with hematoxylin and eosin (H&E), and examined using light microscopy.

### Data analysis

All results are expressed as mean ± SE. The data were compared using ANOVA, followed by Dunn’s multiple comparisons test as post-hoc analysis. A *p* value less than 0.05 was considered to be significant.

## Results

**Figure 1:**
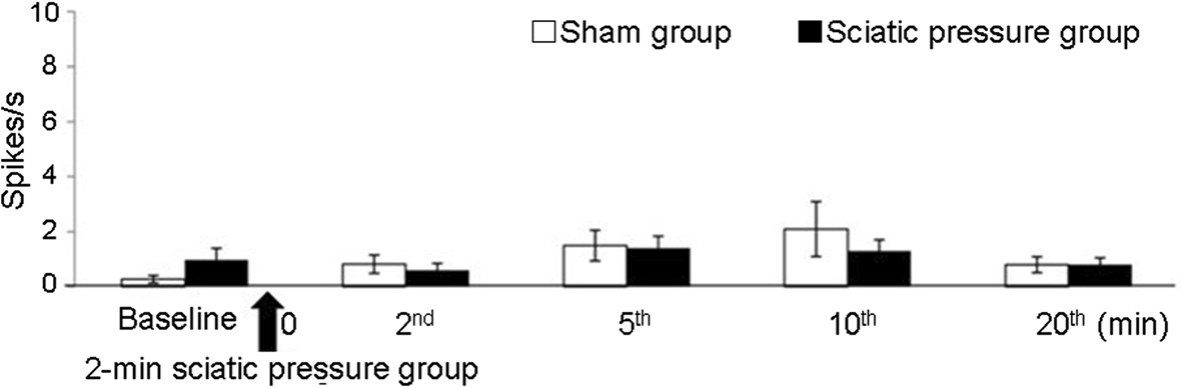
**Spontaneous discharges for the sciatic pressure and sham groups.** The time when the vascular clip was attached to the sciatic nerve is indicated by the black arrow. The “0” point represents the time at which the 2-min period of sciatic nerve pressure was terminated, and at which the post-pressure measurements of spontaneous discharge activity commenced (measured at different time points).

A total of 41 WDR neurons were recorded in the L4-L6 spinal cord region, 30 in the sciatic pressure group and 11 in the sham treatment group. Figure 1 shows the mean spontaneous discharge rate at each time point (baseline, 2^nd^ min, 5^th^ min, 10^th^ min, and 20^th^ min). The average spontaneous discharge rate of the five time points was 1.0±0.2 Hz for the sciatic pressure group, and 1.1±0.3 Hz for the sham treatment group.

**Figure 2:**
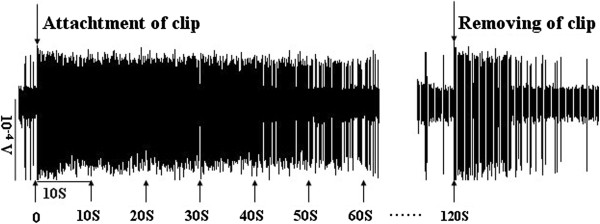
WDR neuron firing caused by the application of a vascular clip to, or removal of it from the sciatic nerve.

Figure 2 illustrates WDR neuronal firing caused by application of a vascular clip to the sciatic nerve, and removal of the clip. Clip attachment triggered short periods of initial firing in 26 WDR neurons, of which 18 exhibited brief continuous repetitive firing after the initial firing burst. Thereafter, the firing rate of these neurons returned to pre-attachment levels. In the remaining 4 neurons, neither initial firing nor continuous repetitive firing was observed. Removal of the vascular clip produced initial firing in 21 neurons, of which 2 exhibited continuous repetitive firing after initial transient firing. In the remaining 9 neurons, neither initial firing nor continuous firing was observed.

**Figure 3:**
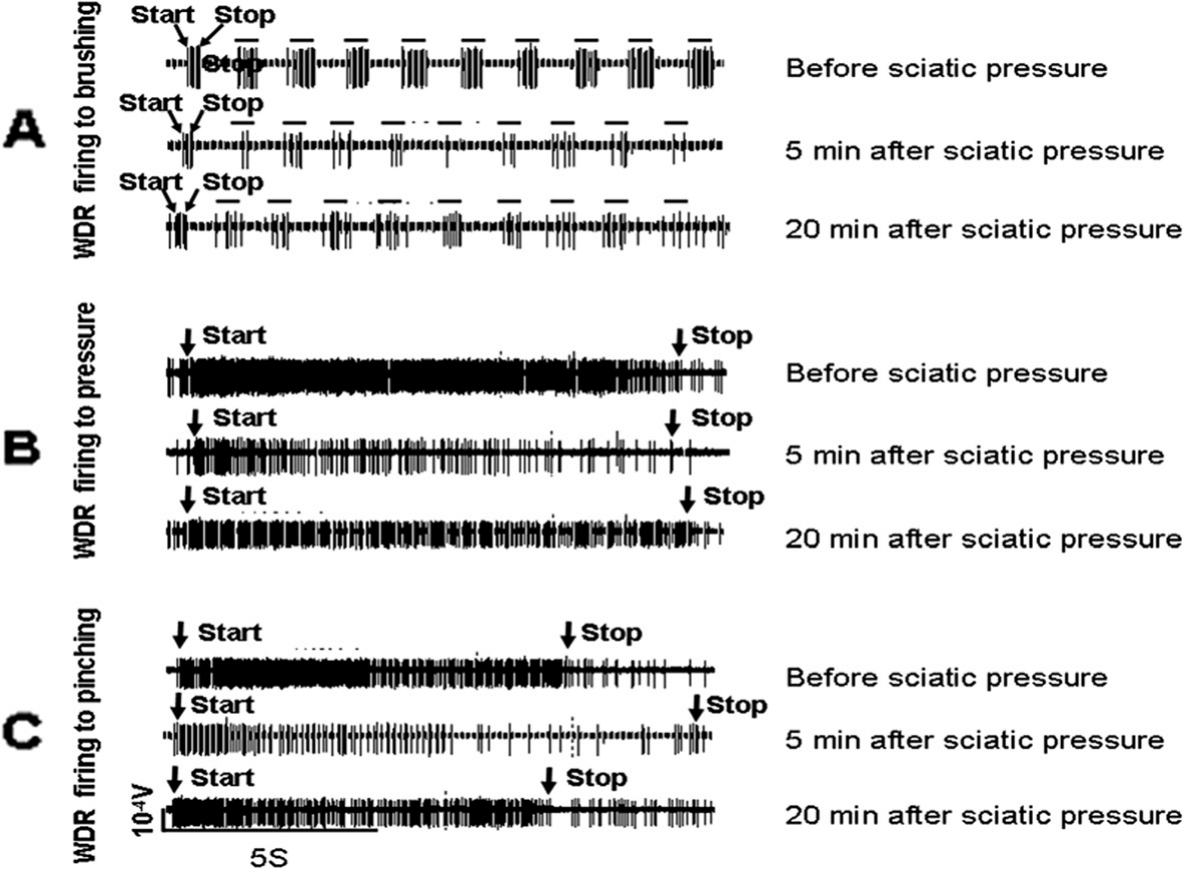
**An example of the response of a WDR neuron to the three stimuli.** The response of a WDR neuron to brushing (**A**), pressure (**B**) and pinching (**C**) stimulation of the ipsilateral hind paw was recorded before, and 5 and 20 min after cessation of a 2-min application of pressure to the sciatic nerve. A horizontal bar in Figure 3A indicates firing associated with each application of brushing.

Figure 3 shows an example of the response of a WDR neuron to the three different stimuli (brushing, application of pressure and pinching of the skin) applied to the ipsilateral hind paw before, and 5 and 20 min after release of the 2-min pressure on the sciatic nerve. This pressure applied to the nerve caused rapid inhibition of WDR neuronal firing in response to all three types of stimulation. WDR responses to pressure and pinching recovered 20 min after clip removal, but recovery of the responses to brushing were slower. Some firing associated with each application of brushing at 5 minutes or 20 minutes could be due to spontaneous activity. We implemented a stimulus duration of 10 seconds to the cutaneous receptive filed. But we could not manually control the time precisely in the experiment. Consequently, there was considerable variation in the duration of stimulus application of pressure and pinching (Figure 3).

**Figure 4:**
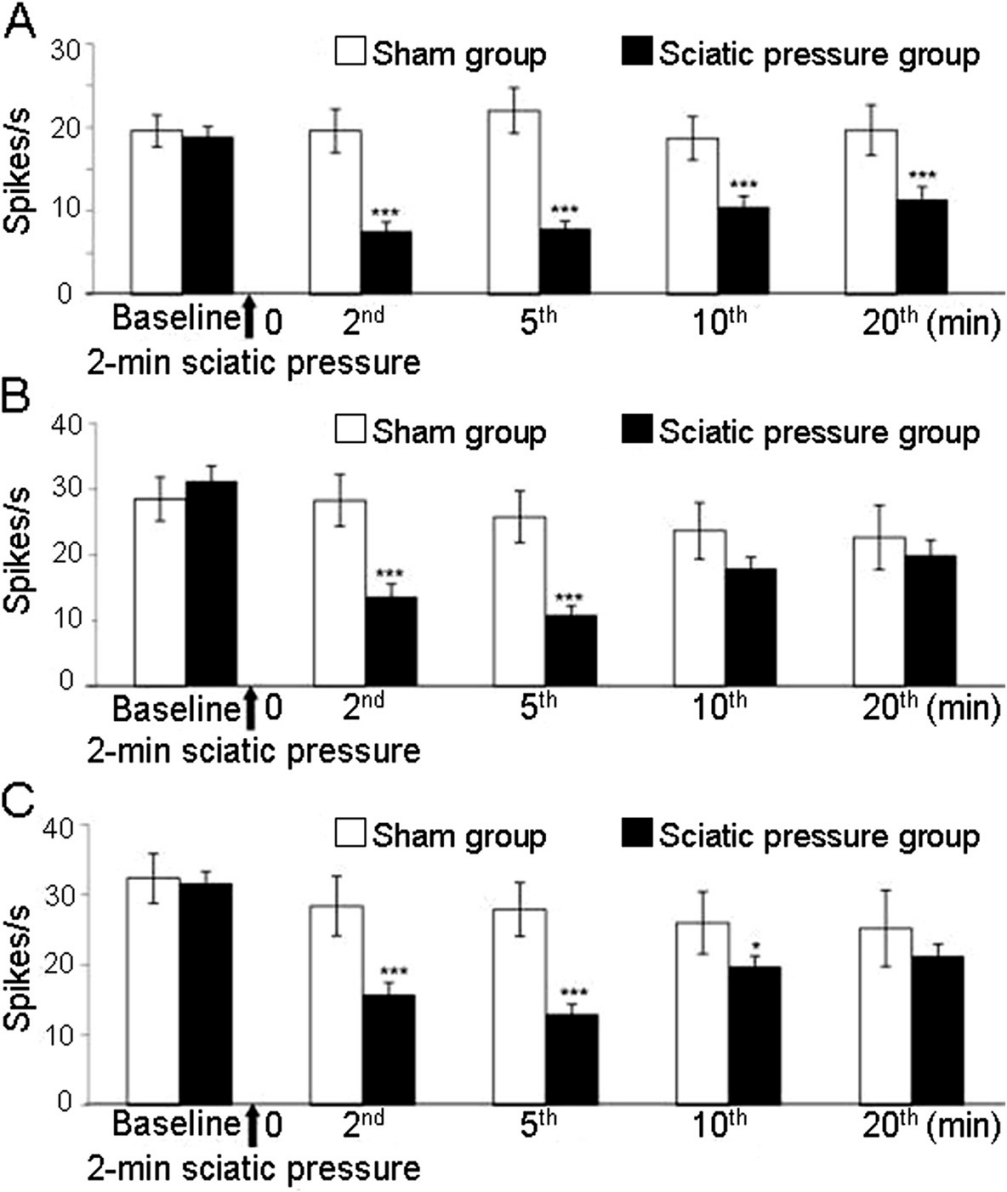
**Time course of the mean firing rate of WDR neurons.** The mean firing rate of a WDR neuron was evoked by application of brushing (**A**), pressure (**B**) and pinching (**C**) stimuli of the skin of the ipsilateral hind paw before, and 2, 5 and 20 min after release of 2-min sciatic nerve pressure. The time that the vascular clip was attached to the sciatic nerve is indicated by the black arrow. Each point (bin) was an average of 11 nerves in the sham group, and 27 nerves in the sciatic pressure group. *p < 0.05, ***p < 0.001, compared with the mean rate of the response before the pressure (sciatic pressure group).

The WDR response to all applied stimuli to the cutaneous receptive filed decreased rapidly after the 2-min application of sciatic nerve pressure in our experiments (Figure 4). The greatest attenuation of the WDR response to pressure (Figure 4B) and pinching (Figure 4C) occurred 5 min after the release of pressure on the sciatic nerve. The mean firing rate, evoked by pressure, decreased from 31.2±2.3 Hz to 10.9±1.4 Hz (*p* < 0.001), and that, evoked by pinching, decreased from 31.7±1.7 Hz to 13±1.4 Hz (*p* < 0.001). Thereafter, the WDR response gradually recovered between 5 and 20 min. WDR responses evoked by brushing to the cutaneous receptive filed also decreased rapidly from 18.9±1.2 Hz to 7.6±1.1 Hz after application of the sciatic nerve pressure (*p* < 0.001) (Figure 4A). Thereafter, the WDR response gradually recovered, but the recovery was slower than that evoked by pressure and pinching.

**Figure 5:**
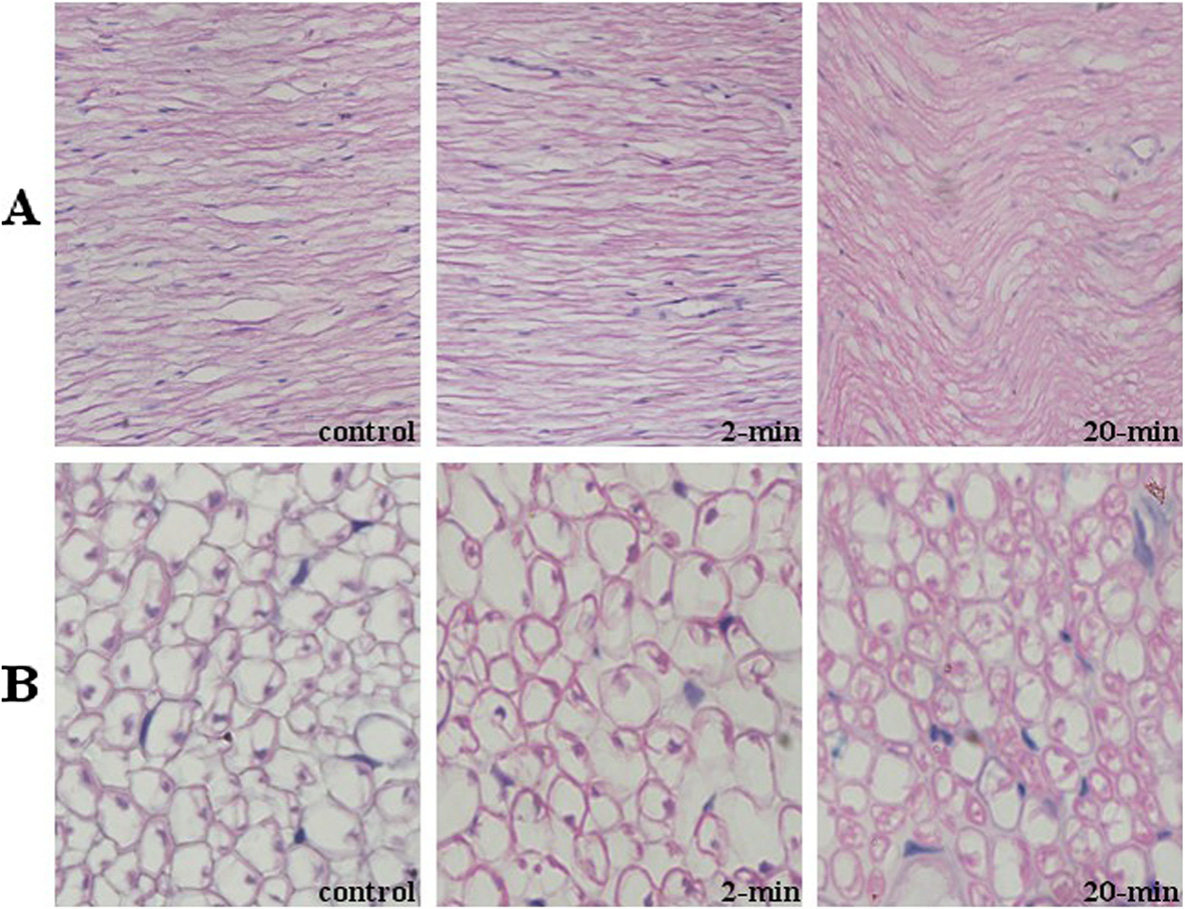
**Intactness of sciatic nerve fibers after the pressure application.** (**A**) longitudinal section of sciatic nerve at the compressed site (original magnification, ×400). (**B**) Cross section of sciatic nerve at the compressed site (original magnification, ×1000). ‘Control’: the sciatic nerve after sham treatment; ‘2-min’: the sciatic nerve after 2 min of pressure at the site; and ’20-min’: the sciatic nerve after 20 min of pressure at the site.

To examine the intactness of the sciatic nerve after 2 minutes of pressure: we compared the histological change after applying pressure to the sciatic nerve for different periods (0, 2, or 20 minutes). Longitudinal or cross sections of the compressed region of the sciatic nerve were prepared and subjected to H&E staining. The differences between the control and the group that received pressure for 2 minutes were not significant. However, a significant difference was observed between the control and the group that received pressure for 20 minutes; the latter group showed an incurved nerve segment in longitudinal sections (Figure 5). Consistently, the cross sections showed that the nerves had narrowed in comparison to the controls. These results are in accordance with previous observations that long-term pressure has a profound effect on nerve dysfunction [21-25].

## Discussion

Studies on the effects of mechanical pressure on nerves have employed different force levels (from several grams to hundreds of grams), for various time periods (from tens of minutes to several weeks), and have demonstrated that pressure duration has a profound effect on nerve function and viability [21-27]. For example, Fern and colleagues studied changes in nerve conduction caused by the deformation and ischemia induced by compression of the cat sciatic nerve [22]. These researchers recorded unitary action potentials from a dorsal root filament during stimulation of the flexor digitorum longus nerve when the sciatic nerve was subjected to 70 mmHg of pressure. Little change was found in conduction of action potentials over the first 19 min of pressure. However, the second, third and fifth fastest action potentials disappeared when the duration of pressure was extended to 28 min, and even the fastest action potential was blocked by 48 min of pressure [22]. These data are in accordance with clinical observations showing that prolonged pressure (even at very low levels) applied to nerves can cause severe neural dysfunction [6-11].

In our previous clinical studies, we showed that manual pressure applied through the skin, soft tissue and muscle on the back of the leg to the sciatic nerve provides significantly more relief from pain than placebo pressure on the front of the leg. We also examined pressure on different parts of the leg, and demonstrated that effective pressure on any accessible area along the sciatic nerve will give rapid pain relief; and the effectiveness is reduced if the same manual pressure is applied at a distance from the sciatic nerve tract [4,13]. These previous clinical results may suggest that, in addition to activating mechanosensitive receptors in the skin and muscle afferent neurons, pressure on the sciatic nerve itself may provide significant analgesic effect. In the present study, we demonstrate that a short duration of pressure (2 min), directly on the sciatic nerve, causes rapid inhibition of WDR responses to both innocuous and noxious mechanical stimuli, and that the inhibitory responses recover within tens of minutes after pressure release. Thus, this animal data may partially explain the rapid analgesia in the clinical setting.

The Diffuse Noxious Inhibitory Control (DNIC) model has been frequently used for quantifying central sensitization in several pain conditions. DNIC relies on painful conditioning stimulation of one part of the body to inhibit pain in another part; the inhibition of pain is rapid and short lasting [28,29]. In our model, pressure on the sciatic nerve is stimulation from another part of the body, and the consequent inhibition induced by the stimulation is rapid and short lasting. Thus, the DNIC mechanism may explain the early inhibition caused by acute pressure on the sciatic nerve in our model.

Sensory transduction in nerves is accomplished by proteins in the membrane called ion channels, which are gated pores that allow the exchange of ions across the cell membrane. Acid-sensitive ion channels (ASIC) have been found expressed in neurons of the mammalian central and peripheral nervous systems, and proposed to constitute mechanoreceptors, and play an important role in responses to mechanical stimuli [30-35]. After a comparison study between ASIC1 knockout mice and wild-type mice for visceral mechanosensation, Page *at al* found ASIC1 contributed to visceral but not cutaneous mechanoreceptor function, and suggested that mechanosensory function in different tissues may involve different mechanisms (33). In a recent report, mice with simultaneous disruptions of ASIC1a, ASIC2, and ASIC3 genes showed increased paw withdrawal frequencies when mechanically stimulated with von Frey filaments. Moreover, in single-fiber nerve recordings of cutaneous afferents, mechanical stimulation generated enhanced activity in ASIC triple-knockouts mice compared to wild-type mice (32). Mogil *et al.* reported ASIC3 mice with a dominant-negative mutation were more sensitive to a number of modalities including mechanical pain, mechanical hypersensitivity after zymosan inflammation, and mechanical hypersensitivity after intramuscular injection of hypotonic saline (36). Four ASIC proteins (ASIC1, ASIC2, ASIC3, and ASIC4) have been found expressed in the sciatic nerve [36]. Thus, rapid inhibition of WDR responses to mechanical stimuli in our model may involve ASICs.

Reports on WDR responses to mechanical stimuli after application of pressure to nerves have been inconsistent. This may be attributable to differences in experimental conditions. The surfaces of the clips that compressed the sciatic nerve were covered with a soft layer of rubber in our studies, which not only absorb a considerable proportion of the pressure, but also protect the nerves from direct damage that might be caused by the clip. The actual pressure on the sciatic nerves estimated varied between 30 and 70 g. This pressure may be several times higher than that employed in clinical studies. Hanai *et al.* used a clip similar to ours to compress the dorsal root or the dorsal root ganglion; the WDR responses to mechanical stimuli increased after release of the pressure [37]. These data differ from ours. The clip force in their study was 40 g; thus, the pressure on the sciatic nerve was similar to ours; but the pressing surfaces of their clips did not have a soft rubber layer. Using clips similar to ours, Kawasaki and *et al.* applied much longer time pressure (30 min), higher pressure (120 g), and without any soft layer on the surface of clips [38].

The WDR responses to pinch and pressure stimuli gradually recovered in both our and Kawasaki’s studies after the release of the pressure on the sciatic nerve. However, in Kawasaki’s study, the WDR response to an innocuous stimulus (brushing) did not show any recovery for 30 minutes after the release of the pressure on the sciatic nerve. In contrast, in our study, the WDR response to brushing, gradually recovered from 7.5 spikes/s to 11 spikes/s within 20 minutes after release of the pressure. Innocuous sensations are mainly mediated by large myelinated afferent fibers (Aβ fibers), which are sensitive to pressure. Whereas, noxious sensations are mediated by fine afferent fibers (Aδ and C fibers) which are sensitive to oxygen deprivation [22,39]. Thus, the lack of recovery of WDR neurons to the brushing stimulus after the release of pressure on the sciatic nerve in Kawasaki’s study may be attributed to damage to large myelinated afferent fibers. Gradual recovery of WDR neurons after the release of pressure in our model, which is similar to but slower than the recovery after release of pinch and pressure, may indicate that nerves were only partially injured, if at all. This is consistent with the histological data: when the injury of sciatic nerves was observed, it occurred when pressure was applied to the sciatic nerve for 20 minutes, but not when the pressure was applied for 2 minutes.

## Conclusions

Acute pressure applied to the sciatic nerve exerts a rapid inhibitory effect on the WDR response to both noxious and innocuous stimuli. Our results may, in part, explain the rapid analgesic effect of acute sciatic nerve pressure noted in clinical studies, and suggest a new model for the study of pain.

## Competing interests

The authors declare that they have no competing interests.

## Authors’ contributions

JH and JC are the primary investigators in this study. WW and WT were responsible for the animal experiments and drafted the manuscript. DL, JL, YY, QW, WZ and BW helped or were responsible for part of the experiments. All authors have read and approved the final manuscript.
